# Supplementary material for: A long noncoding RNA HILinc1 enhances pear thermotolerance by stabilizing PbHILT1 transcripts through complementary base pairing
Source: Commun Biol. 2022 Oct 26;5:1134. doi: 10.1038/s42003-022-04010-7 (PMC9606298; doi:10.1038/s42003-022-04010-7)

Supplementary Table 1 LncRNAs differently expressed under 38 °C treatment.

Supplementary Table 2 Mass spectrometry results of semi-*in vivo* pulldown assay by PbHILT1-GST

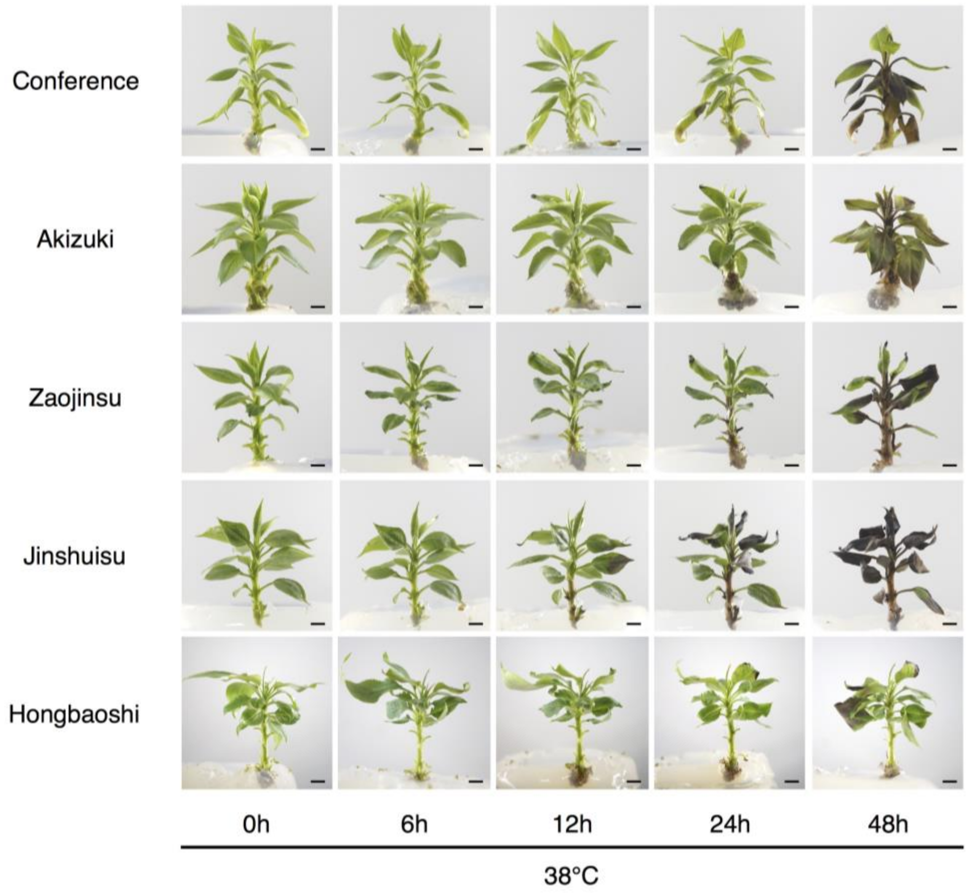

Supplementary Figure 1 Phenotype changes of different pear species under 38 °C. Bars = 0.5 cm, five plants of each genotype were used for phenotype observation and the representative images are shown.

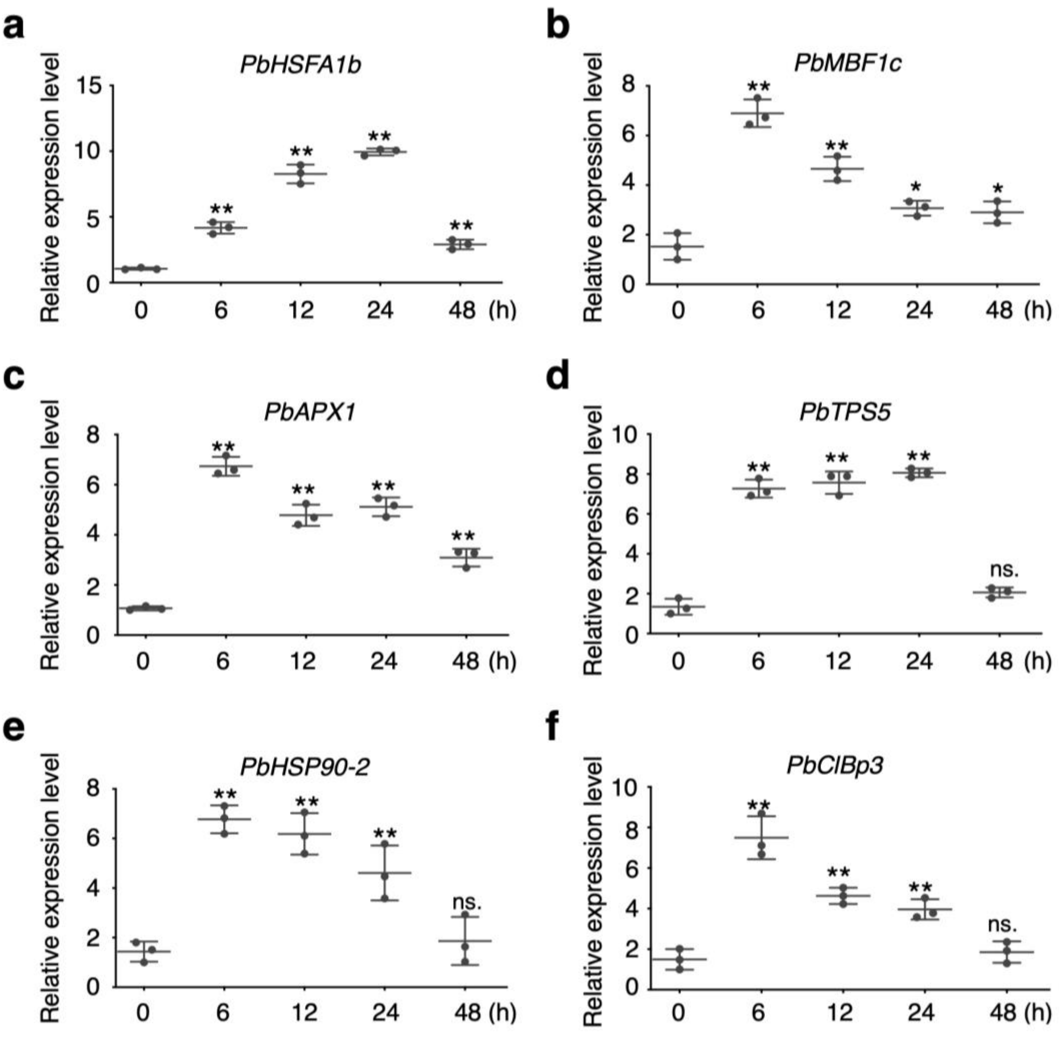

Supplementary Figure 2 Relative expression levels of heat shock response genes in 'Hongbaoshi' exposed to 38 °C for different periods by RT-qPCR. Error bars represent the mean ± SD (n=3). Significant differences were determined by two-tailed student's t-test (*P < 0.05, **P < 0.01).

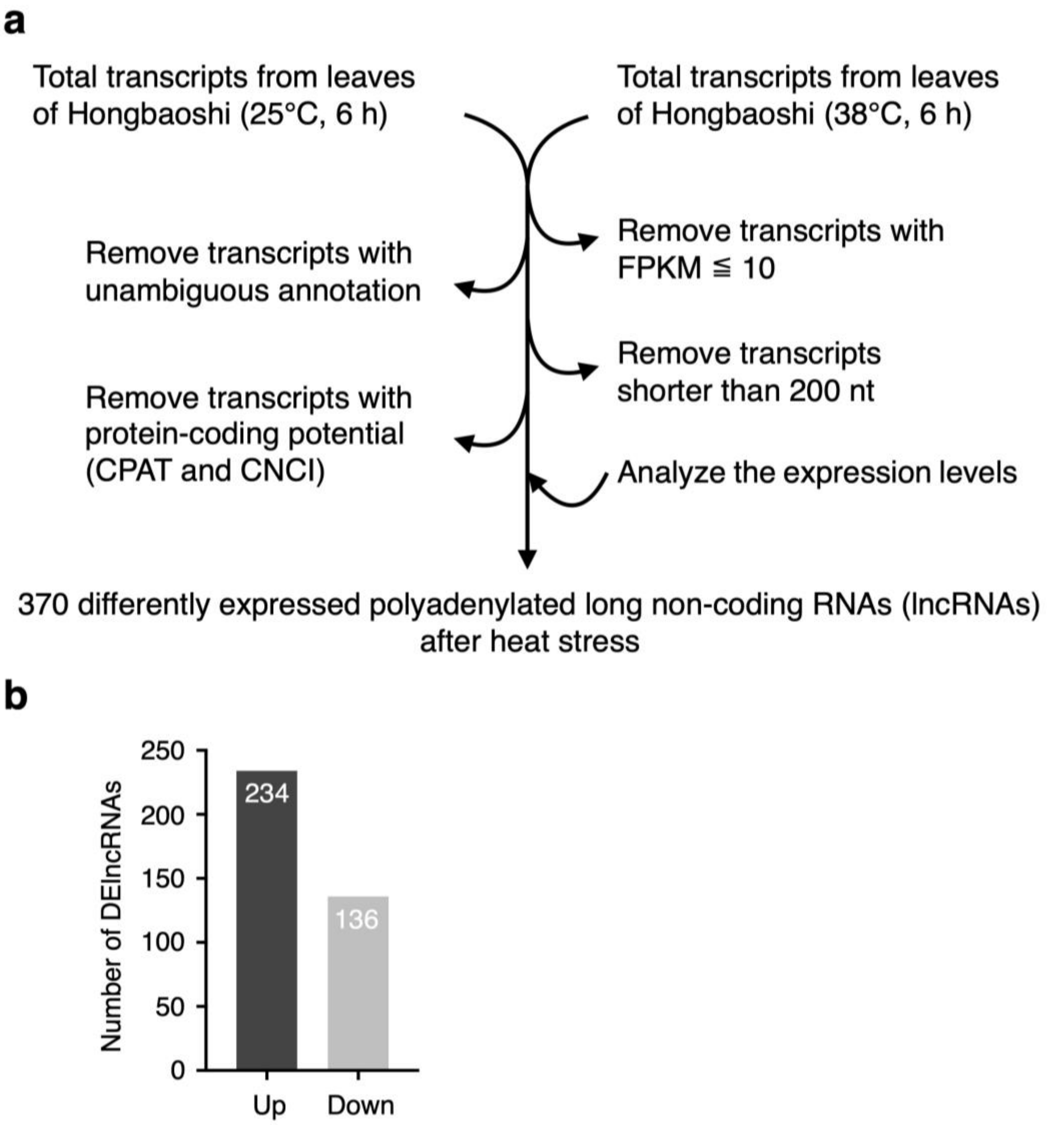

Supplementary Figure 3 Analysis of differently expressed lncRNAs after heat stress. **a** Pipeline for differently expressed lncRNA analysis at 38 °C compared to 25 °C. **b** Numbers of upregulated and downregulated lncRNAs after heat stress.

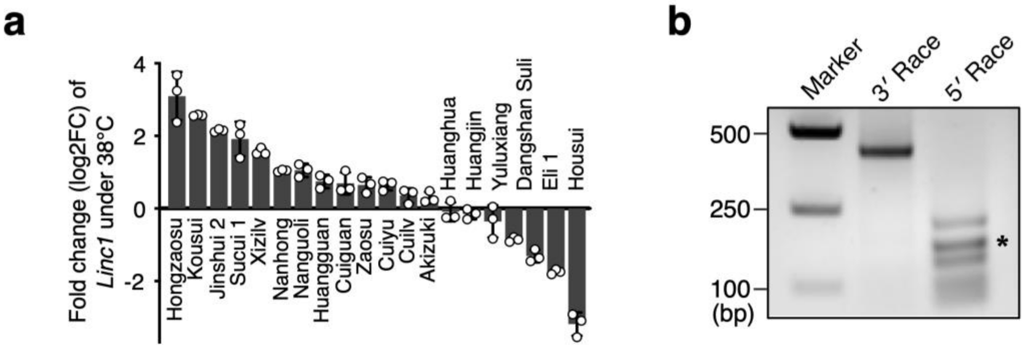

c

CACTTCAATGATTCATTTACTTTGTGTACAGCACTCTTGCTATGTTAATATGTTCGCTTAAGACATG
TAACGATGGCCTCTTGCATTCGAATCTTACATGAGCCCAATTCGATGGATCATAATGTATTGATTT
TCTTCGTATTTATTTTTGATGGACGAGTAAAGTTACA

5′ race nested reverse primer 2

CGTACGATTATACCAGAAGATTTCTACTTCTTTAGGCTATGGTTTGTAAAAACTTGCATCCTCTAA
ATTGCTTAATCATTTGTTTTGAACTATTTTTCTAGCAATTAAAAGCATATTATAGCCGTGTTTAAGAC
GAAATCTAAAAGTGATCTTATATATATAAACCGACCCTAGCTCATACCAGCAAATTGGGCAGATAT
AAAAAGTTTGAGGTGCCCCAATTTTTAATGGAGTAGGATTCTCTTTCCTCTTTTTTCCCTCCTCTT
TTCTCTCCTCTTATTTGAACGGTCACAGTTAAGCCACGTCAACATCTTATATTAATTTTTTATAGAA
AGAGAAAAACGAAATAAAGAATGTGAGAGAAAGAGATGAAAGATGATGAAAAGAAAAGGGGAG
AGAATCCTAATTCATTTTTAACATGATTATGTTTCATCAAAGTTATGCTGCCCCAACTCTTCTGTG
GAAAGTATTCAGTTTGTTGAATCTTGATGCAAACGCGATTTCATTTGTTGCCATTTTGTTCTTTTA
TTTTCCCACTCGTACTACATTGCACTGCACGACAATTTGTACTACATCTTGCGCGTATACCTGTA
CTAAATTCCAGTACTCCAACAACCTGGCTTATTTTCCTGTACTGGATTTTGGTACTCGAACCTTG
GTTATTATTGATTCAGAAATATATATACGTGTGTGTATATGGATCATCAGTATATGGATAGTTGCAGC
AGAAATCTTTCAGTTTAACAGGAGAATCACACAGACGCTCCTCTACTCCAACTTCAAGAATTGG
AGGCGATGCAGTCCATTCTTATGGAGTGAGTTCATCTTTACTGTTCGATCTAATGAATCTATATGA
ATTTATGGCATCCACAATTGGCACCAAATTCATCACCAGAGTGCTACCTTTCTCACCTTCAGCTT
CGAAGTCTTCCAACAAGTCCCTTGTCCTTGACCTCATGCTACTACTTCGATCTCGTTTCTTATGT
TAGTTATATGCAGGCCATGCATAAACAAAACCGGGCCGGCCATTTTTCATACCTTGGCACCAAA
GTCACCACCCGACTGTTATCGTATTCAATTCATCCTCCAACAAGTCCGTGATCTCTATGCGCAAT
CTCTTAATTTATGATAAAATCAACAAAGAAATGCTATGAAAAGAAAGTAGTTTGCGGATAGAGATA
ACGAACTTGTCGTAGGATGAGCTGAAGATGATAACAGTCTGGTGGTTACTTTGGT

3′ race nested forward primer 2

GCCAAGGCATACAAGATGGTCACCTTATTTTACTTTTTTTCAGAAATCAAAGATCTTATGTAACAA
ATTAACTAAAAATGCAAATAGTTGATTTATTTACTCACTTTCTAATGTTCCTCTGATATATATTGTGA
AAAATATTAGTGTGTATATTAAAATGTATCAGCATCATCTTATGGCAACATCAGCCAATAATAAGGT
TTTTCAATCGTATAAGAAACCATCTACAATCAAGACAAAGTTGGCAATGATAATGATTTAATCCATA
AAATTAGGATAACTATCAACGCAATTAAAGGCCTTTGCCAGTTCACTTGTAACATCTTGTTTTTTT
CGAACTAGTAAACGTTGACAGATAGTTTCCATTACAATTGACTTCATGGTAAAAGTAAATGTCAAA
ATAGATTCAGCTAGCAACCAAATGGACCAGCACATCACTTGTCA

Supplementary Figure 4 Full-length sequences of heat-responsive *Linc1* obtained by 3' and 5' RACE. **a** Detection of *Linc1* expression 6 h post heat treatment in different pear species by RT-qPCR. Error bars represent the mean ± SD (n=3). Significant differences were determined by two-tailed student's t-test (*P < 0.05, **P < 0.01). **b** PCR products of 3' and 5' RACE detected by agarose gel electrophoresis. Asterisk represents the specific band of 5' RACE products. **c** Sequences of 5' (in blue) and 3' (in red) ends of *Linc1* transcript according to sequencing results.

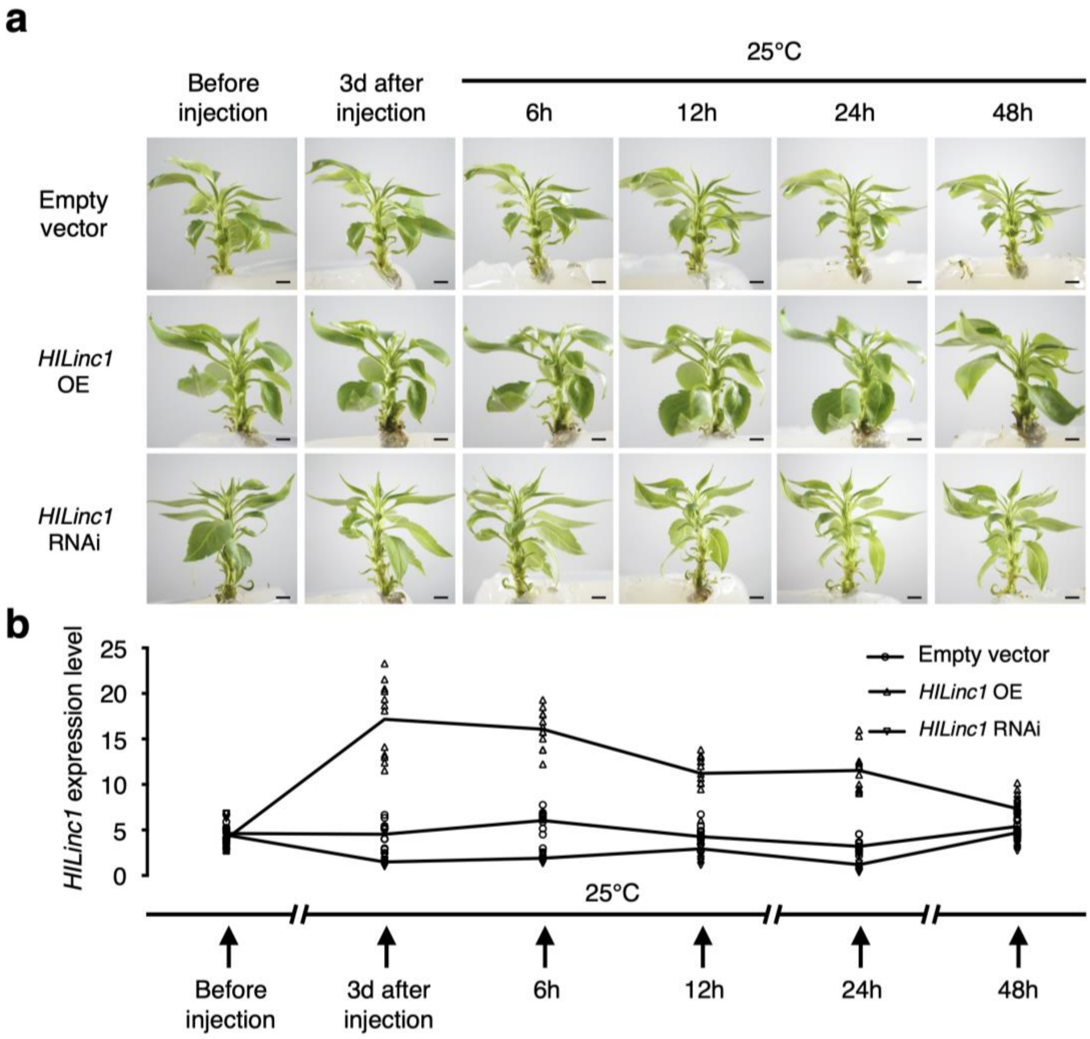

Supplementary Figure 5 Regulation of *HILinc1* has no influence on phenotype of pear under normal condition. **a, b** Phenotype (**a**) and *HILinc1* expression level (**b**) of *HILinc1* overexpressing (OE) and silencing (RNAi) lines under normal condition (25 °C), lines expressing empty vector serves as controls. 'Hongbaoshi' pears vacuum-infiltrated with *p35S::HILinc1*, *p35S::RNAi-HILinc1* or empty vector were treated with 25 °C 3 d after transformation. Leaves before and 3 d after infection (0 h post treatment), 6, 12, 24, 48 h post treatment were harvested for RT-qPCR analysis. In **a**, bars = 0.5 cm, ten plants of each genotype were used for phenotype observation and the representative images are shown.

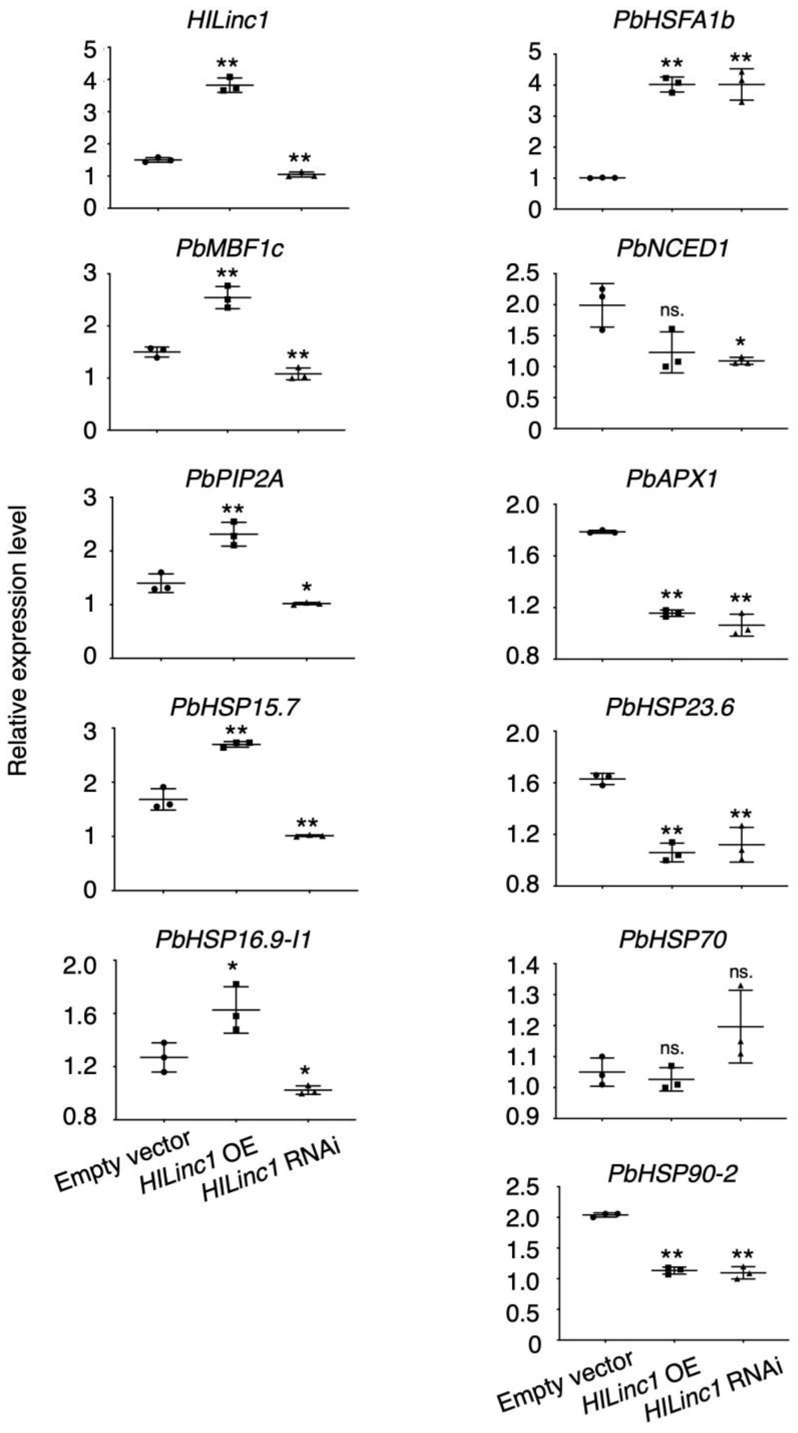

Supplementary Figure 6 Heat maps showing the relative expression level of ten selected heat shock response genes in control, *HILinc1* OE and RNAi lines under 38 °C for 6 h by RT-qPCR. The experiments were performed independently for three times.

a

ATGGTGACAGGCCGCTGGACATTCCTTGGCACCAAAGTTACCTTAATACTGCCATCGTCGTCAG
CTCCCAAGTCCTCCAACAAGTCCGTAATCCCAAGATGCAGGCTGGTTTTAACCTTCTTCTGGAA
GTGTGGCAAATACACCGAGCTCCTTGCTAACTCGGACTTGTCGGGTCGACTCAGCGAGTCCTC
GTCATCATTTACATGCACGTCGAACTTGAGAGCCTCATCCCCACAAACTCAATCCCTTCAATCAC
CAACACCTTCTCTTCCTCCTCCTTGTCTCACCTCCGAGTCCAAAGCTATTGGCAACTTACTACTC
TCAACCAAAACTACCTATTGAACAAATATAGTGCATAATTGCTATATTTTAATCACACACATGATTC
AAGAAACTAAAAATATGCAATTTAATTTATGCCCATGAGAAAAACAATGTTAAATAATATAACAAAAT
AGGTTAGAGAAACATGAAAAAAGTTAGAGGCAAATGTTTGACATTTGTTCTTAAGACATTTCACC
ACCTTCTTTGACACCTCTGCCGCATTTGCTGGCCCTGTTTTGTTATGACTCTCCAAGGCAAGTC
CTCCTAGACCAAGGAGAAAGATTTCTCCTATCCAATTTTACTCGACAAGTTTGCGGATCACGATT
AGGGTTTTGGTCACTATTGATTGTTTTTGCTTTCATGAACCTCTAAGATTTCTGACTACGGAAATC
TGGGATTTGTTTGGGAAGATGTGAGGGATTGAAGGGGTGGTGTAGTAGGTGGTAATCATGGTG
TTGGAGGATATAGTGGTGGAGGAGAAGCCACAACTTTGTTTCCATTTTGTCAAGAGCTATGTATC
CAGATCCCTTTTCATCAAATTCATCTAGTTCGAGGATTCAGACTGTTGAATTTTAATCAAATGGTT
ACCGTTATTATAACTTTTAAATGTTCATCTTTTTGAATTTAATCAAATGGTTACAGTTATTATAACTTT
TAAATATTCATCTTGTTTGTAATCATTGGATCAAAATCTAACTATCTAAATCTTAGAGTAGGAGAATT
TGGTGAAAAGAGGTATGTATGAACAATTTACGTCAGTTGGAGTGACTAATTACATTTGCAGCCAC
ACGTTCTATGTCACCATGCTTTAGTCAAGATAAGGACGAGACTTTTATAACAGGCCCTACACTTG
TCTAAATCACACC

HSE

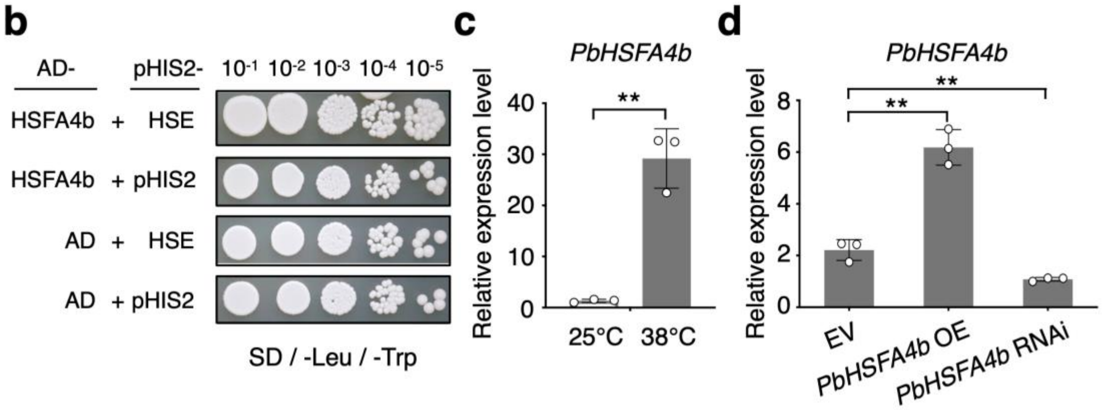

Supplementary Figure 7 PbHSFA4b directly upregulates expression of *HlLinc1*. **a** Sequences of *HlLinc1* promoter region (1190 bp upstream from transcription start site). The 342-bp long ORF is highlighted in red. Heat shock element (HSE) is underlined in blue. **b** Binding of PbHSFA4b to the *HlLinc1* promoter fragment containing the HSE confirmed using yeast one-hybrid assays. **c** Detection of *PbHSFA4b* expression in response to heat by RT-qPCR. **d** Detection of *PbHSFA4b* expression in control, *PbHSFA4b* OE and RNAi lines under 38 °C for 6 h by RT-qPCR. Error bars in **c** and **d** represent the mean ± SD (n=3). Significant differences were determined by two-tailed student's t-test (**P < 0.01).

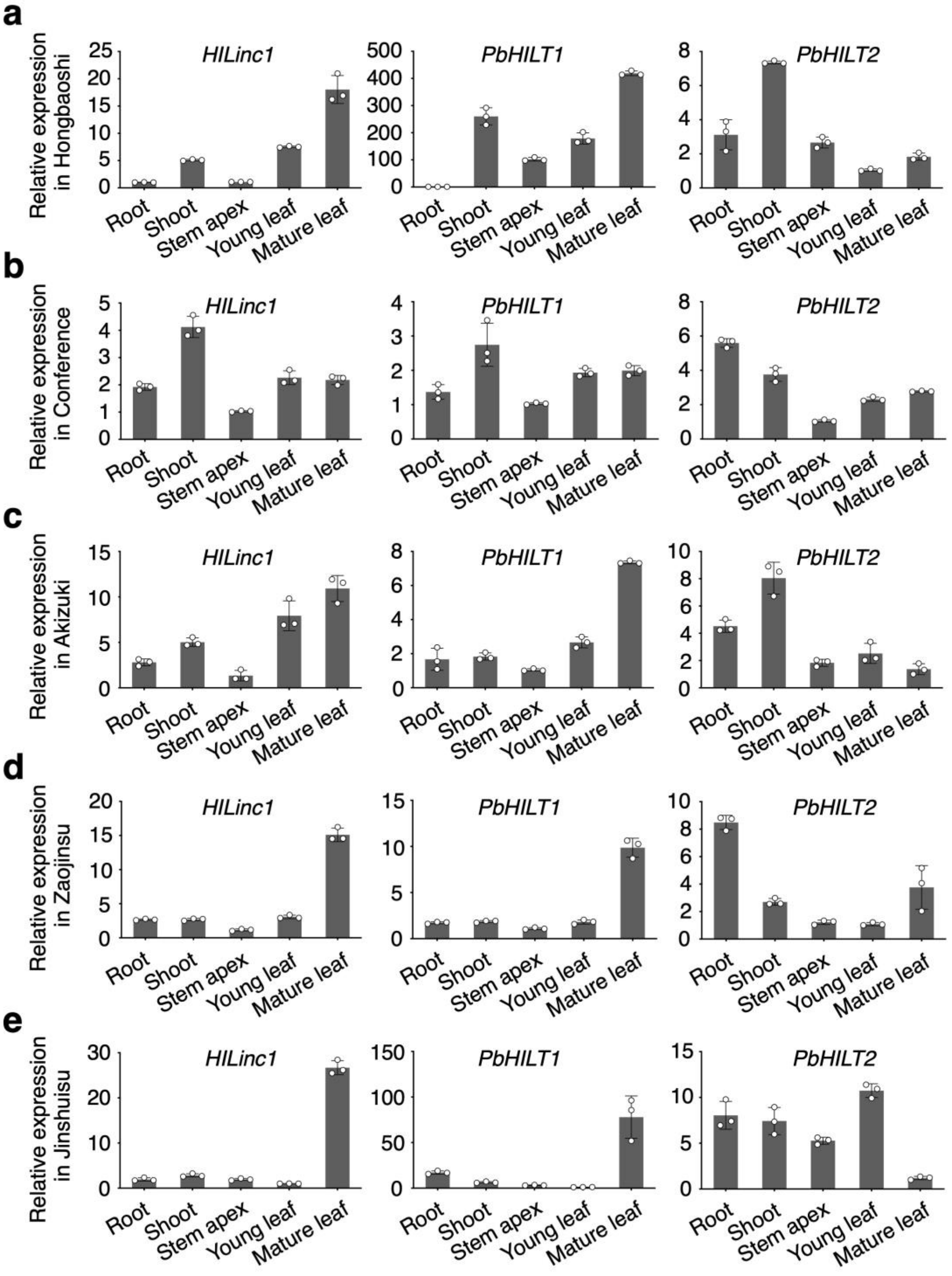

Supplementary Figure 8 Tissue specific expression pattern of *HILinc1*, *PbHILT1* and *PbHILT2* in five tissue-cultured pear species by RT-qPCR. Error bars represent the mean ± SD (n=3).

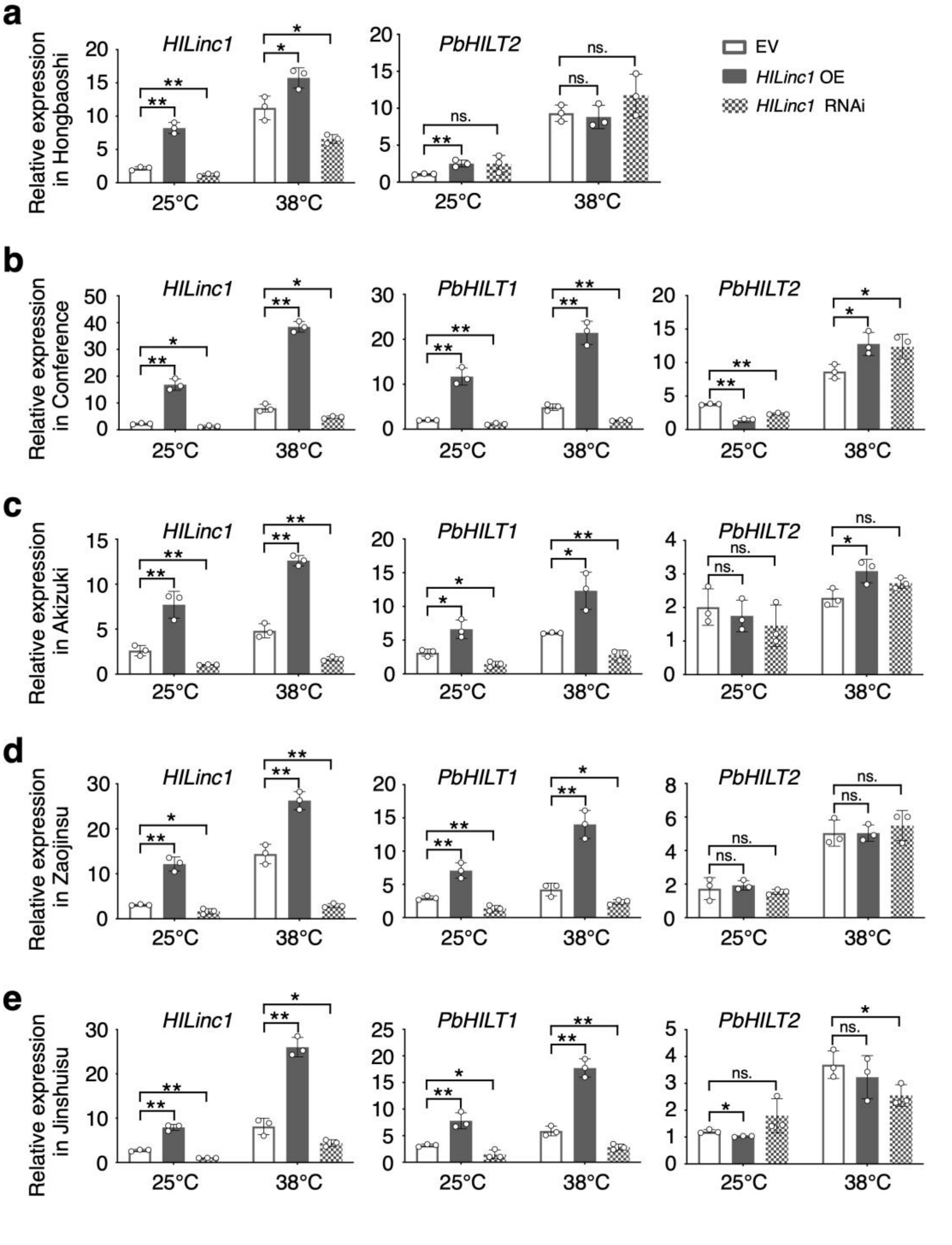

Supplementary Figure 9 Relative expression level of *HlLinc1*, *PbHILT1* and *PbHILT1* in control, *HlLinc1* OE and RNAi lines of different pear species under 25 °C and 38 °C by RT-qPCR. Error bars represent the mean ± SD (n=3). Significant differences were determined by two-tailed student's t-test (*P < 0.05, **P < 0.01).

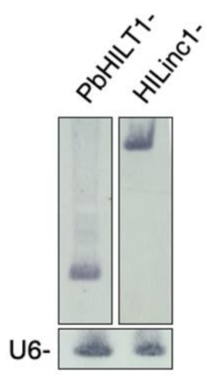

Supplementary Figure 10 Northern blot analysis of the complementary region of *PbHILT1* and *HILinc1*. *PbHILT1-* and *HILinc1-* represented the antisense probe. U6 was used as the internal control.

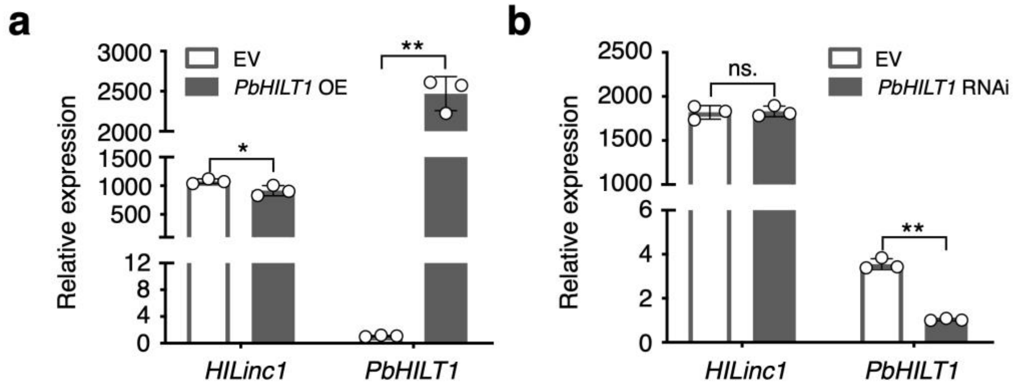

Supplementary Figure 11 Regulation of *PbHILT1* has no effect on *HILinc1*. **a** Detection of *HILinc1* and *PbHILT1* expression in control and *PbHILT1* OE lines under normal condition (25 °C) by RT-qPCR. **b** Detection of *HILinc1* and *PbHILT1* expression in control and *PbHILT1* RNAi lines under normal condition (25 °C) by RT-qPCR. Error bars in **a** and **b** represent the mean ± SD (n=3). Significant differences were determined by two-tailed student's t-test (*P < 0.05, **P < 0.01).

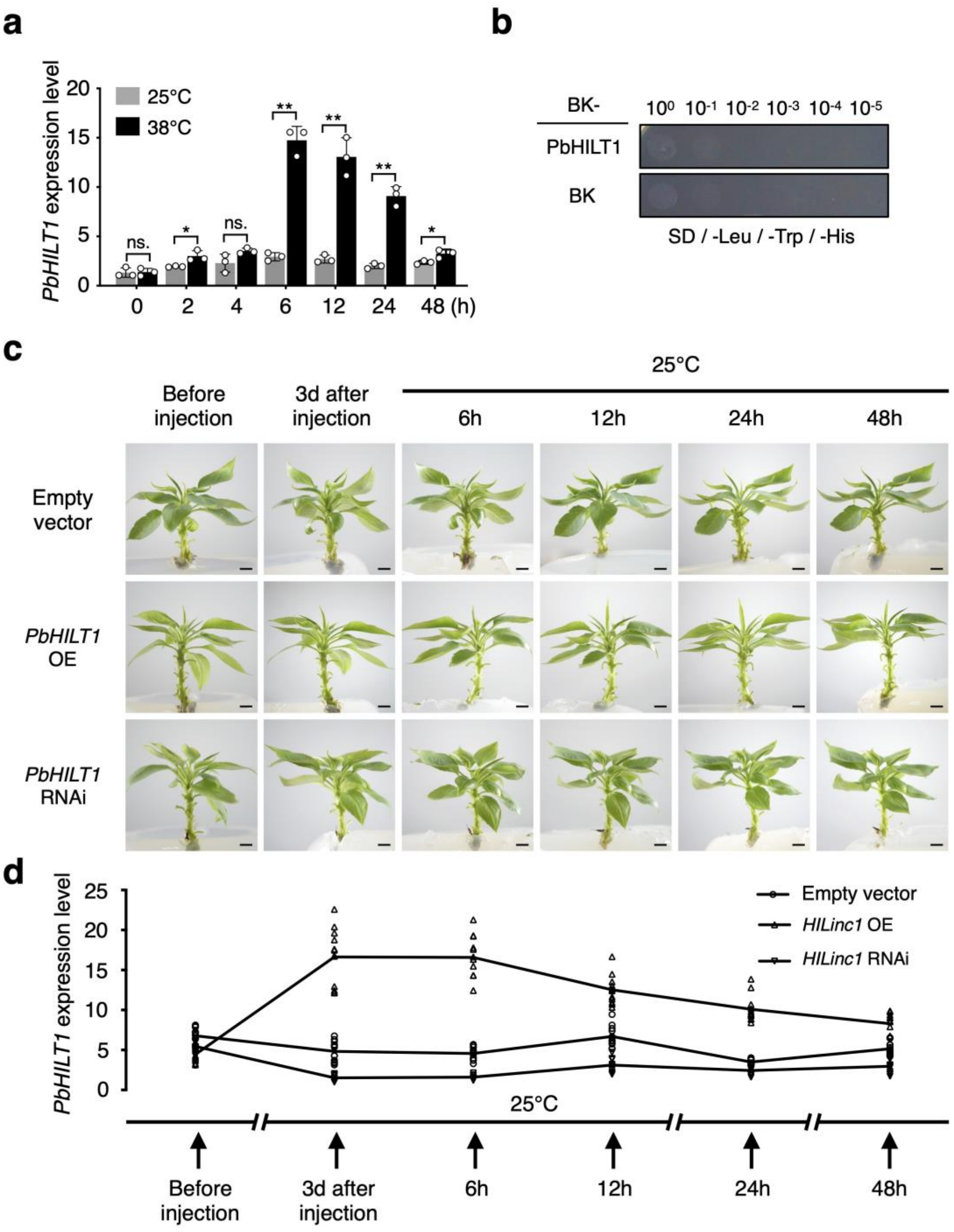

Supplementary Figure 12 Characteristics of *PbHILT1* in pear. **a** Temporal expression pattern of *PbHILT1* in 'Hongbaoshi' under 38 °C by RT-qPCR. Error bars represent the mean ± SD (n=3), significant differences were determined by two-tailed student's t-test (*P < 0.05, **P < 0.01). **b** Self-activation of PbHILT1 in yeast strain AH109. The full-length coding sequence (CDS) of *PbHILT1* was cloned into PGBKT7, and the

recombinant construct was transformed into AH109. Yeast clones were grown on synthetic triple dropout medium (–Leu/–Trp/–His). **c, d** Phenotype (**c**) and *PbHILT1* expression level (**d**) of *PbHILT1* OE and RNAi lines under normal condition (25 °C), lines expressing empty vector serves as controls. 'Hongbaoshi' pears vacuum-infiltrated with *p35S::PbHILT1*, *p35S::RNAi-PbHILT1* or empty vector were treated with 25 °C 3 d after transformation. Leaves before and 3 d after infection (0 h post treatment), 6, 12, 24, 48 h post treatment were harvested for RT-qPCR analysis. In **c**, bars = 0.5 cm, ten plants of each genotype were used for phenotype observation and the representative images are shown. Error bars in **a** represent the mean ± SD (n=3). Significant differences were determined by two-tailed student's t-test (*P < 0.05, **P < 0.01).

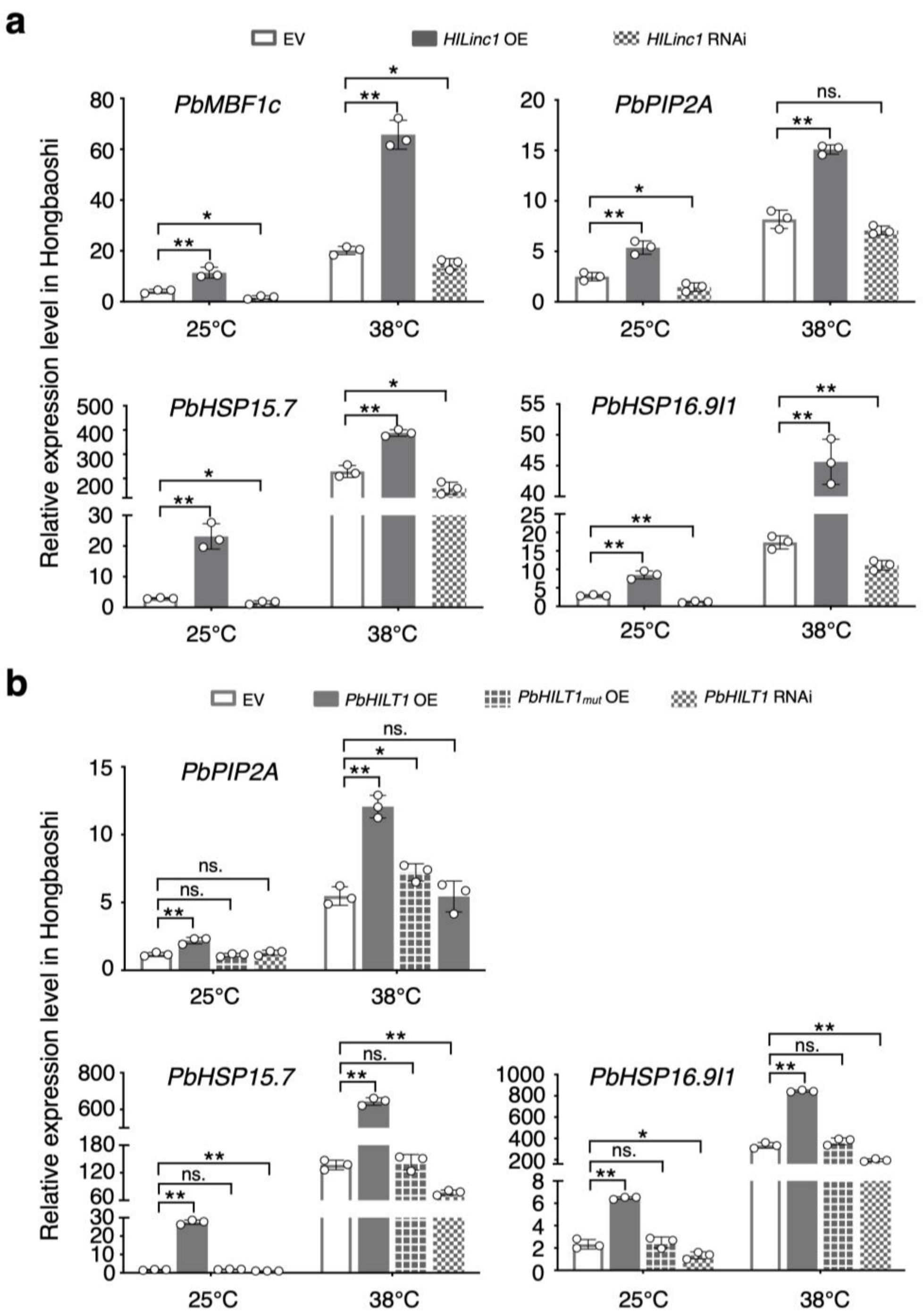

Supplementary Figure 13 Detection of *PbMBF1c*, *PbPIP2A*, *PbHSP15.7* and *PbHSP16.9-I1* expression after *HILinc1*/*PbHILT1* regulation in 'Hongbaoshi' by RT-qPCR. **a** Relative expression level of *PbMBF1c*, *PbPIP2A*, *PbHSP15.7* and *PbHSP16.9-I1* in *HILinc1* OE and RNAi lines. **b** *PbPIP2A*, *PbHSP15.7* and *PbHSP16.9-I1* expression in *PbHILT1* OE, $PbHILT1_{mut}$ OE and *PbHILT1* RNAi lines. $PbHILT1_{mut}$ was generated by an adenine insertion after the initiation codon of *PbHILT1*. Error bars represent the mean ± SD (n=3). Significant differences were determined by two-tailed student's t-test (*P < 0.05, **P < 0.01).

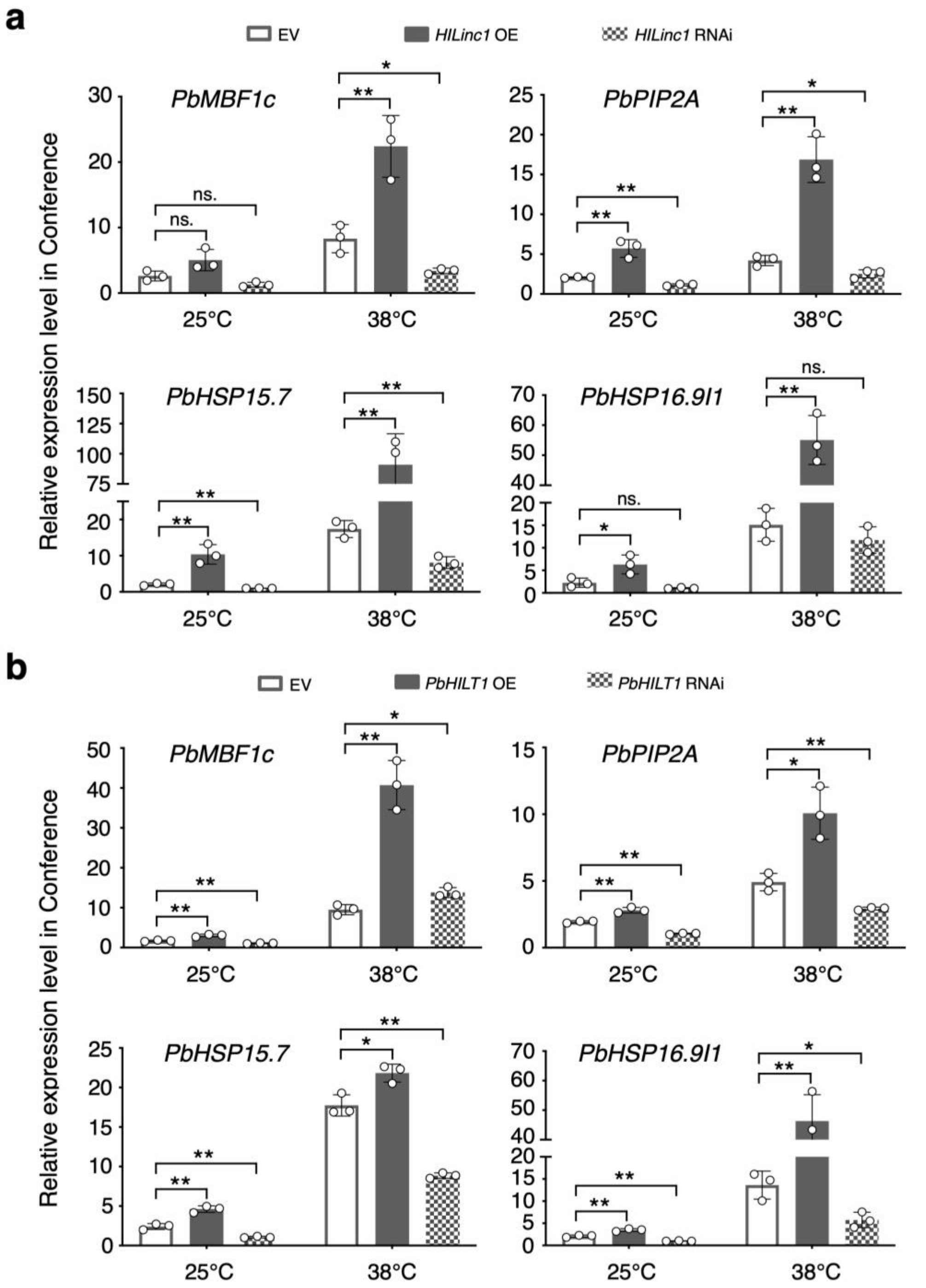

Supplementary Figure 14 Detection of *PbMBF1c*, *PbPIP2A*, *PbHSP15.7* and *PbHSP16.9-I1* expression after *HlLinc1*/*PbHILT1* regulation in 'Conference' by RT-qPCR. **a** Relative expression level of *PbMBF1c*, *PbPIP2A*, *PbHSP15.7* and *PbHSP16.9-I1* in *HlLinc1* OE and RNAi lines. **b** *PbMBF1c*, *PbPIP2A*, *PbHSP15.7* and *PbHSP16.9-I1* expression in *PbHILT1* OE and RNAi lines. Error bars represent the mean ± SD (n=3). Significant differences were determined by two-tailed student's t-test (*P < 0.05, **P < 0.01).

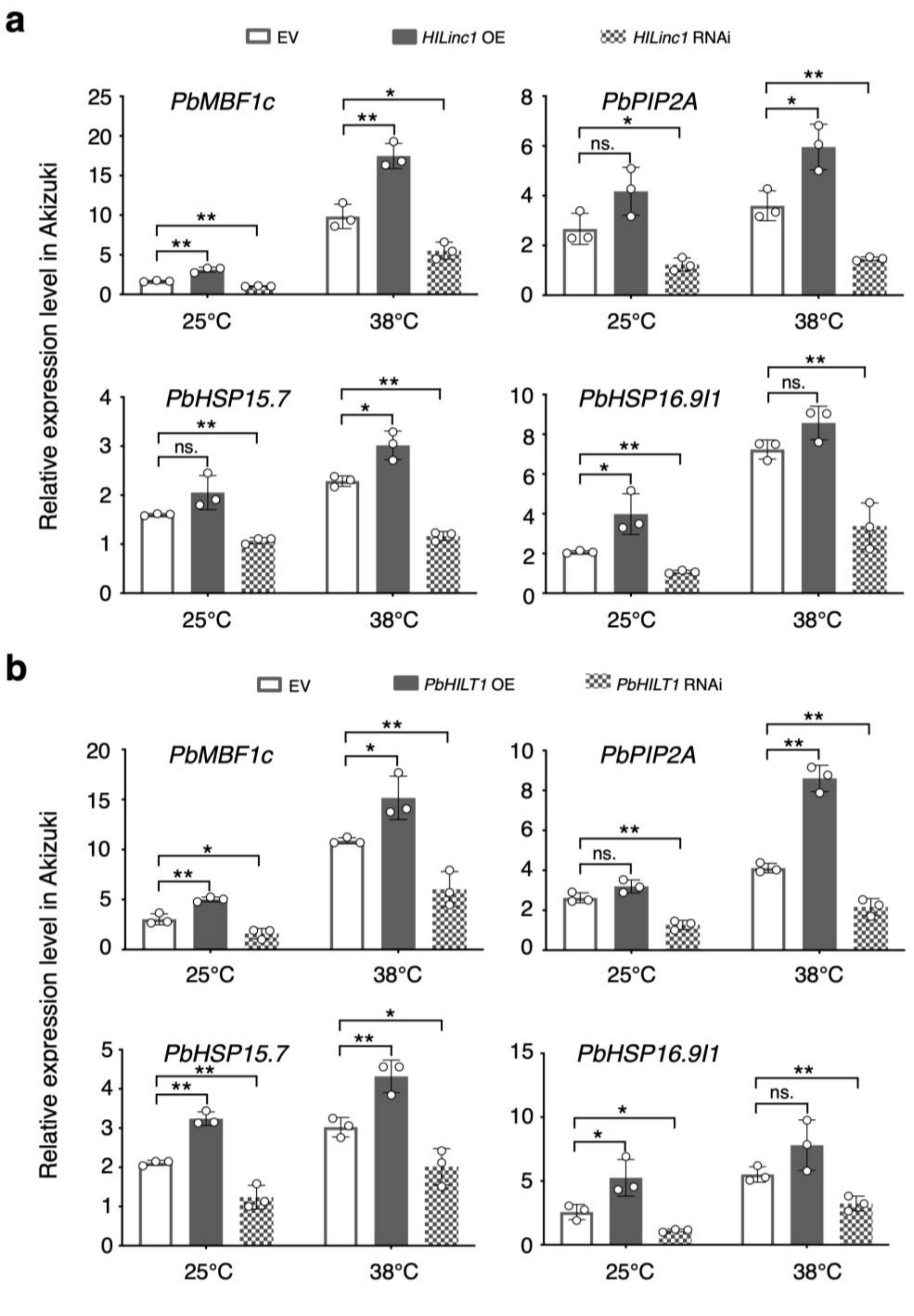

Supplementary Figure 15 Detection of *PbMBF1c*, *PbPIP2A*, *PbHSP15.7* and *PbHSP16.9-I1* expression after *HILinc1*/*PbHILT1* regulation in 'Akizuki' by RT-qPCR. **a** Relative expression level of *PbMBF1c*, *PbPIP2A*, *PbHSP15.7* and *PbHSP16.9-I1* in *HILinc1* OE and RNAi lines. **b** *PbMBF1c*, *PbPIP2A*, *PbHSP15.7* and *PbHSP16.9-I1* expression in *PbHILT1* OE and RNAi lines. Error bars represent the mean ± SD (n=3). Significant differences were determined by two-tailed student's t-test (*P < 0.05, **P < 0.01).

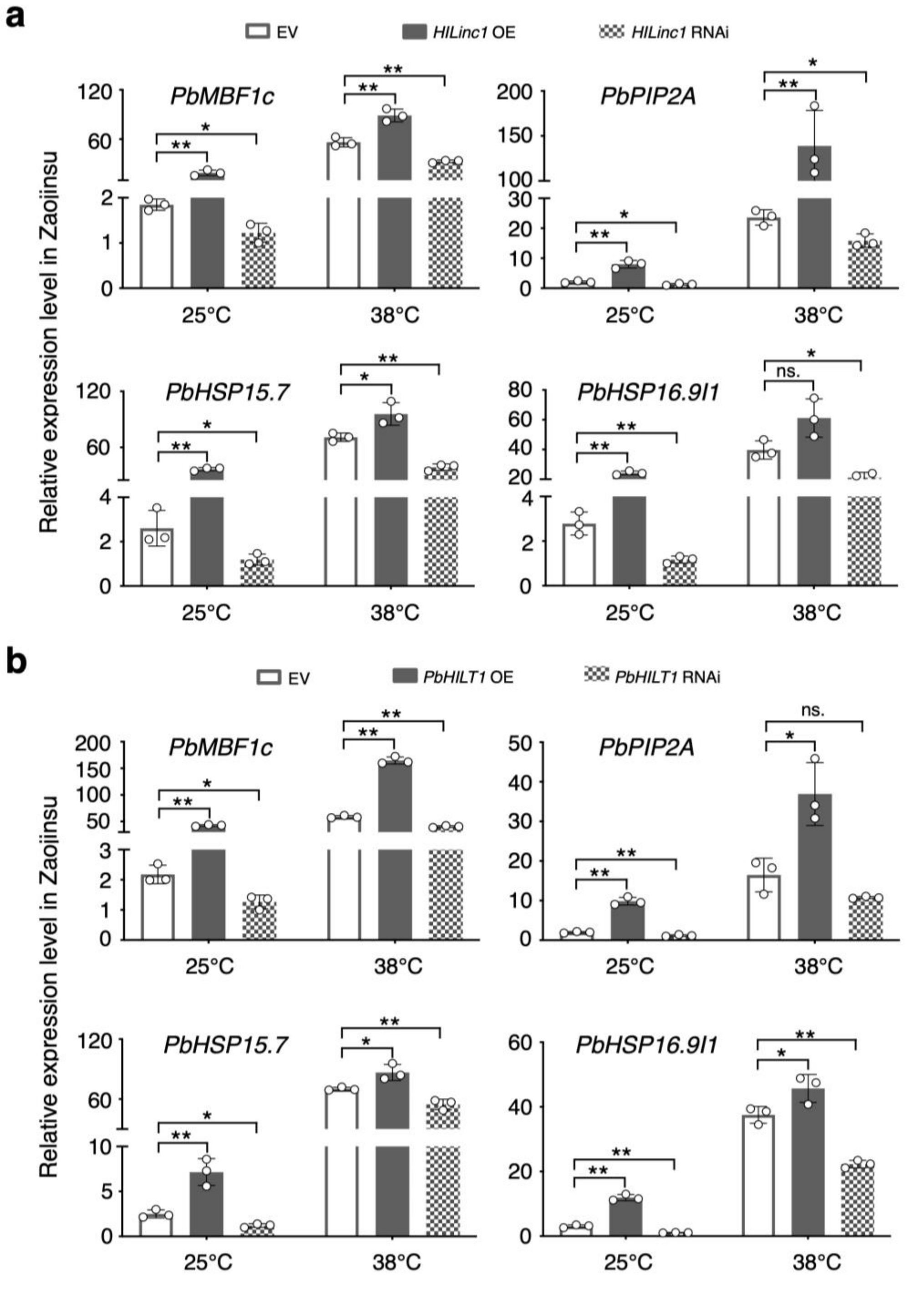

Supplementary Figure 16 Detection of *PbMBF1c*, *PbPIP2A*, *PbHSP15.7* and *PbHSP16.9-I1* expression after *HILinc1*/*PbHILT1* regulation in 'Zaojinsu' by RT-qPCR. **a** Relative expression level of *PbMBF1c*, *PbPIP2A*, *PbHSP15.7* and *PbHSP16.9-I1* in *HILinc1* OE and RNAi lines. **b** *PbMBF1c*, *PbPIP2A*, *PbHSP15.7* and *PbHSP16.9-I1* expression in *PbHILT1* OE and RNAi lines. Error bars represent the mean ± SD (n=3). Significant differences were determined by two-tailed student's t-test (*P < 0.05, **P < 0.01).

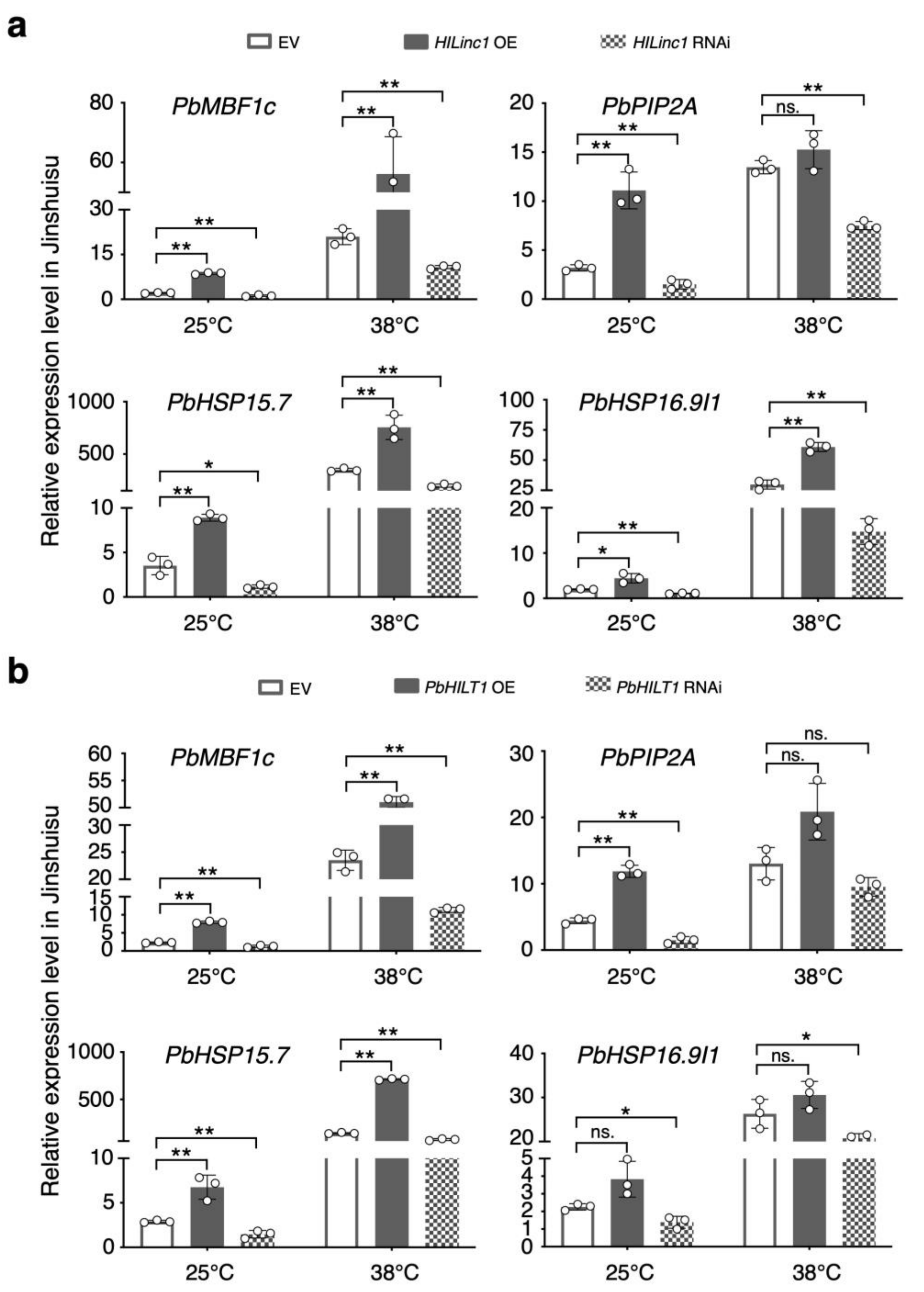

Supplementary Figure 17 Detection of *PbMBF1c*, *PbPIP2A*, *PbHSP15.7* and *PbHSP16.9-I1* expression after *HILinc1*/*PbHILT1* regulation in 'Jinshuisu' by RT-qPCR. **a** Relative expression level of *PbMBF1c*, *PbPIP2A*, *PbHSP15.7* and *PbHSP16.9-I1* in *HILinc1* OE and RNAi lines. **b** *PbMBF1c*, *PbPIP2A*, *PbHSP15.7* and *PbHSP16.9-I1* expression in *PbHILT1* OE and RNAi lines. Error bars represent the mean ± SD (n=3). Significant differences were determined by two-tailed student's t-test (*P < 0.05, **P < 0.01).

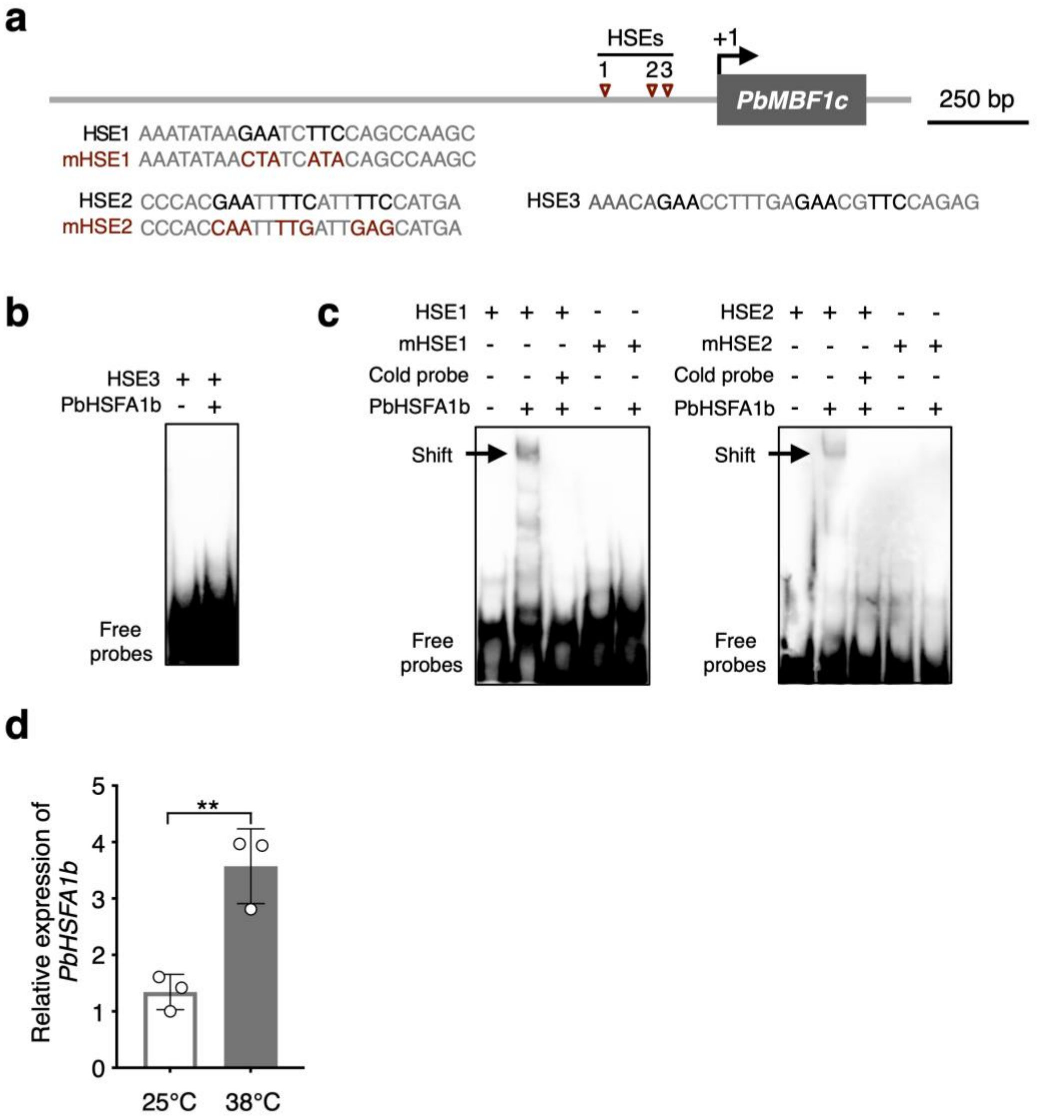

Supplementary Figure 18 PbHSFA1b directly upregulates expression of *PbMBF1c*. **a** Schematic representation of the 3 HSEs on *PbMBF1c* promoter region (1500 bp). Sequences of normal (upper) and mutant (down) form HSEs are highlighted in red. **b** EMSA showing the non-binding of PbHSFA1b to HSE3 on *PbMBF1c* promoter. **c** Electrophoretic mobility shift assays showing the direct binding of PbHSFA1b to HSE1 and HSE2 on the *PbMBF1c* promoter. **d** Detection of *PbHSFA1b* expression in response to heat by RT-qPCR. Error bars represent the mean ± SD (n=3). Significant differences were determined by two-tailed student's t-test (*P < 0.05, **P < 0.01).

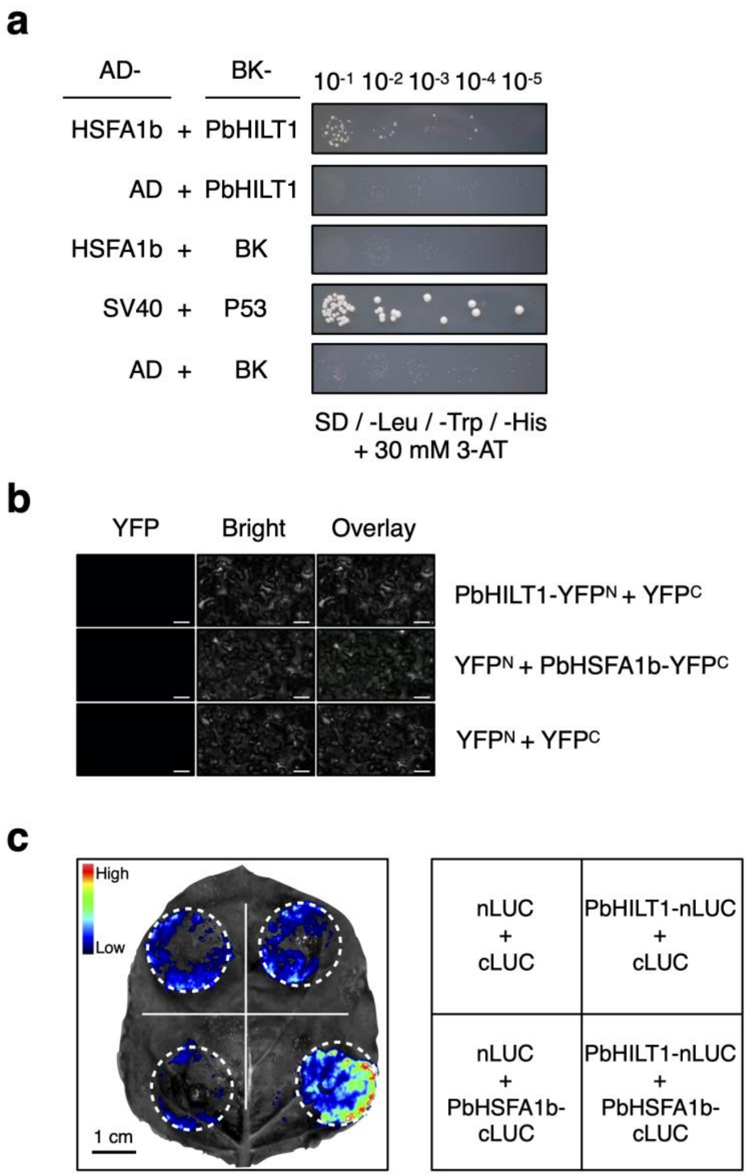

Supplementary Figure 19 PbHILT1 interacts with PbHSFA1b. **a** Interaction between PbHILT1 and PbHSFA1b, as determined in yeast two-hybrid assays. **b** Confocal images of negative controls in BiFC assays. **c** Split-luciferase assays in tobacco leaves. Images were captured 48 h after *Agrobacterium* infiltration. Bar = 1cm. The experiments were performed independently for three times and the representative images are shown.

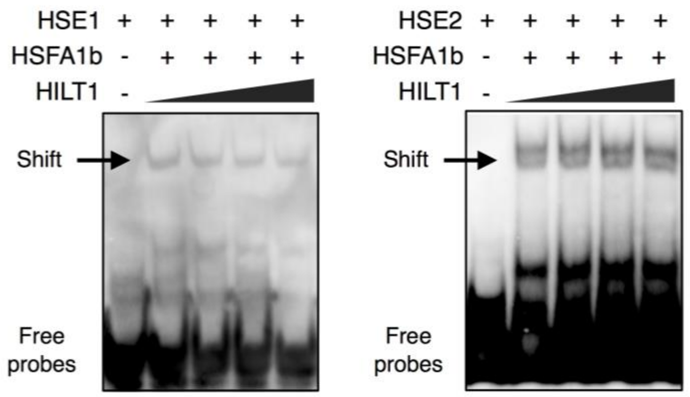

Supplementary Figure 20 PbHILT1 shows no influence on the binding of PbHSFA1b on *PbMBF1c* promoter in EMSA assays.

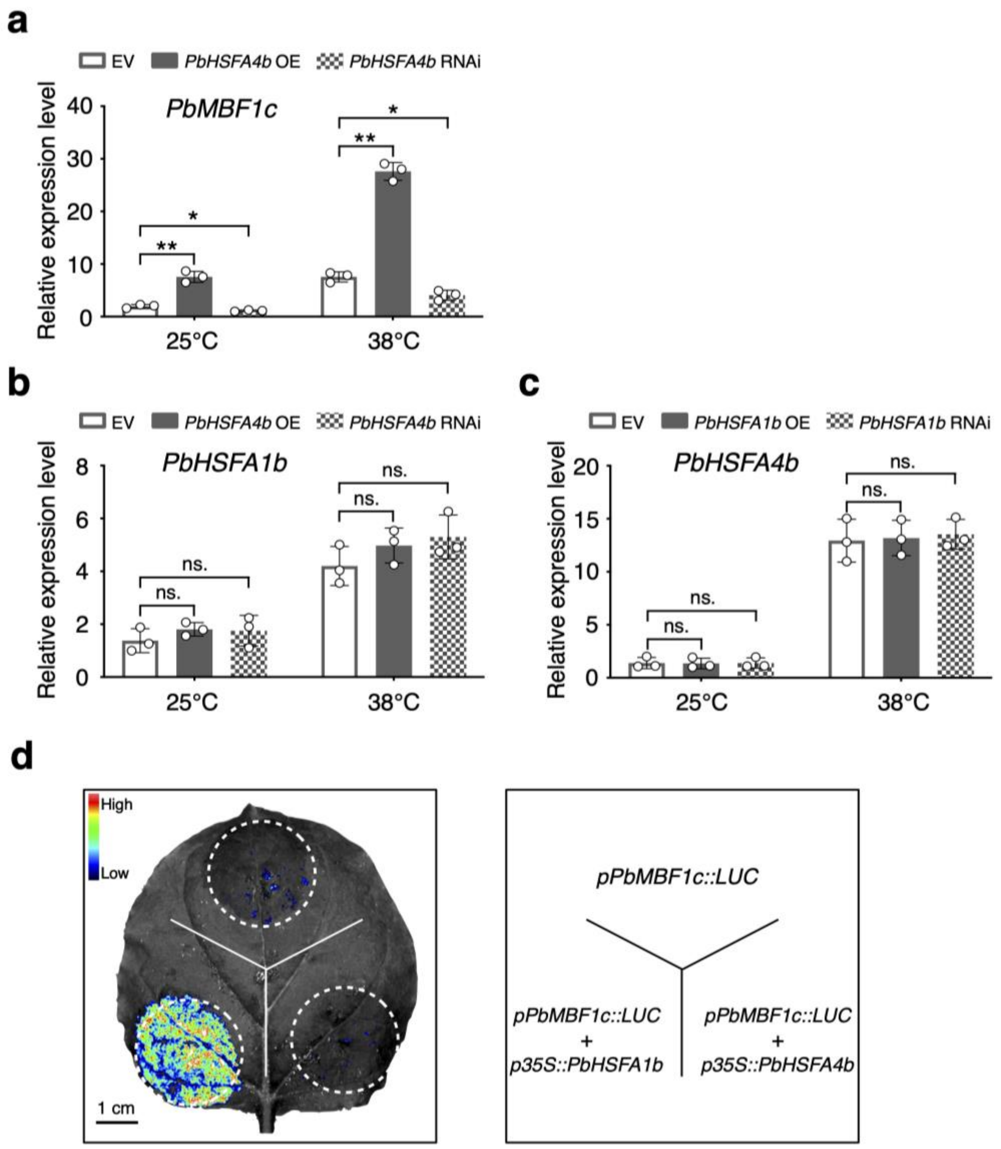

Supplementary Figure 21 The transcriptional regulation between *PbHSFA4b*, *PbHSFA1b* and *PbMBF1c*. **a** Detection of *PbMBF1c* expression in control, *PbHSFA4b* OE and RNAi lines under 25 °C or 38 °C for 6 h by RT-qPCR. **b** Detection of *PbHSFA1b* expression in control, *PbHSFA4b* OE and RNAi lines under 25 °C or 38 °C for 6 h by RT-qPCR. **c** Detection of *PbHSFA4b* expression in control, *PbHSFA1b* OE and RNAi lines under 25 °C or 38 °C for 6 h by RT-qPCR. Error bars in **a**, **b** and **c** represent the mean ± SD (n=3). Significant differences were determined by two-tailed student's t-test (*P < 0.05, **P < 0.01). **d** *pPbMBF1c::LUC* was co-expressed with *p35S::PbHSFA4b* and *p35S::PbHSFA1b* respectively in tobacco leaves. Split-LUC assays were performed 48 h after *Agrobacterium* infiltration. Bar = 1cm. The experiments were performed independently for three times and the representative images are shown.

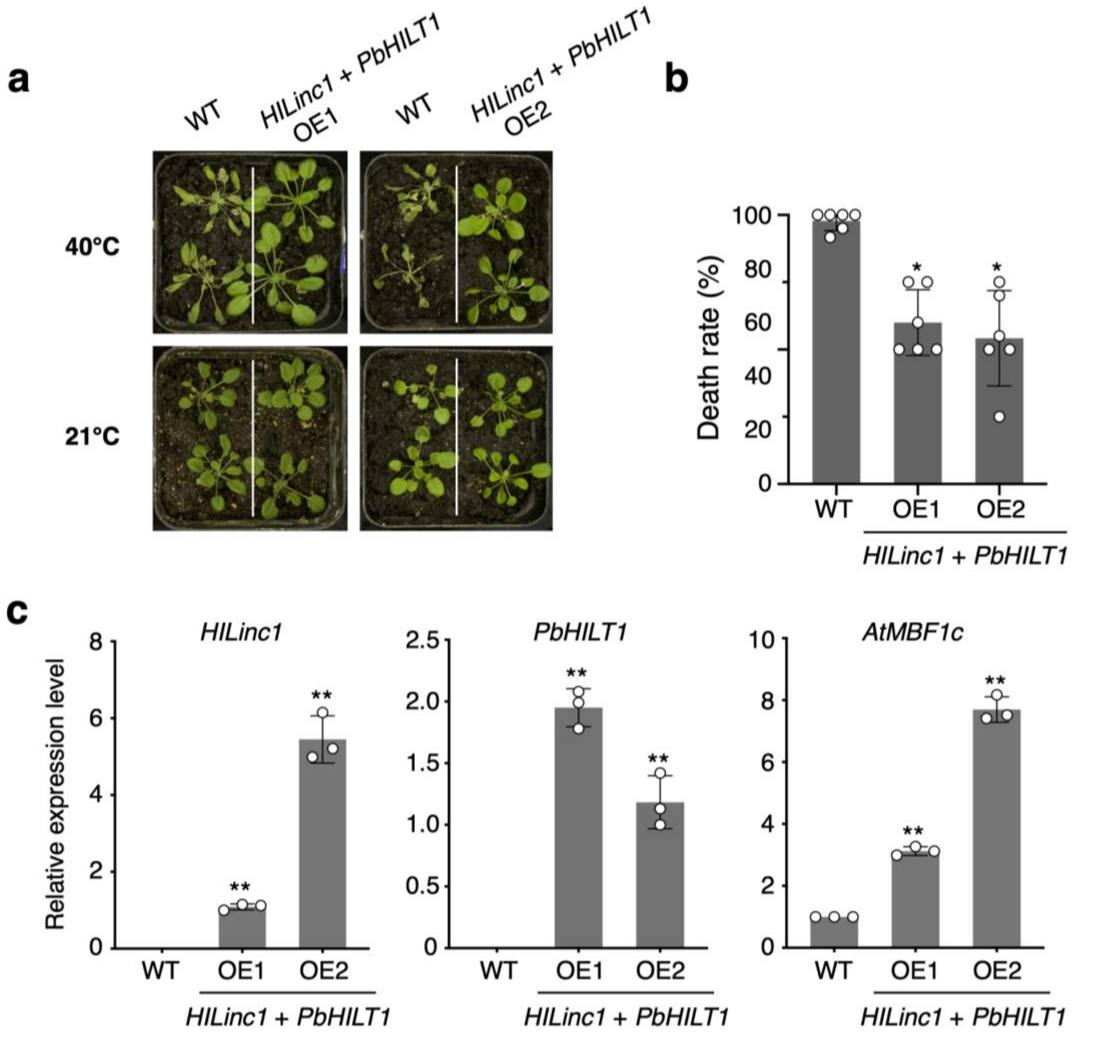

Supplementary Figure 22 *HILinc1* and *PbHILT1* transformation enhances thermotolerance in *Arabidopsis thaliana*. **a** Phenotypes of representative *HILinc1*/*PbHILT1* plants (T2) after 40°C treatment for 4 days, followed by recovery under 21°C for 7 days. **b** Death rates of *HILinc1*/*PbHILT1* and wild type plants in **a**. Error bars represent the mean ± SD (n=3), 20 plants were calculated for each replicate. **c** Relative expression level of *HILinc1*, *PbHILT1*, and *AtMBF1c* in *HILinc1*/*PbHILT1* plants. Error bars represent the mean ± SD (n=3). Significant differences were determined by two-tailed student's t-test (*P < 0.05, **P < 0.01).

Unprocessed images in figures

Figure 1c

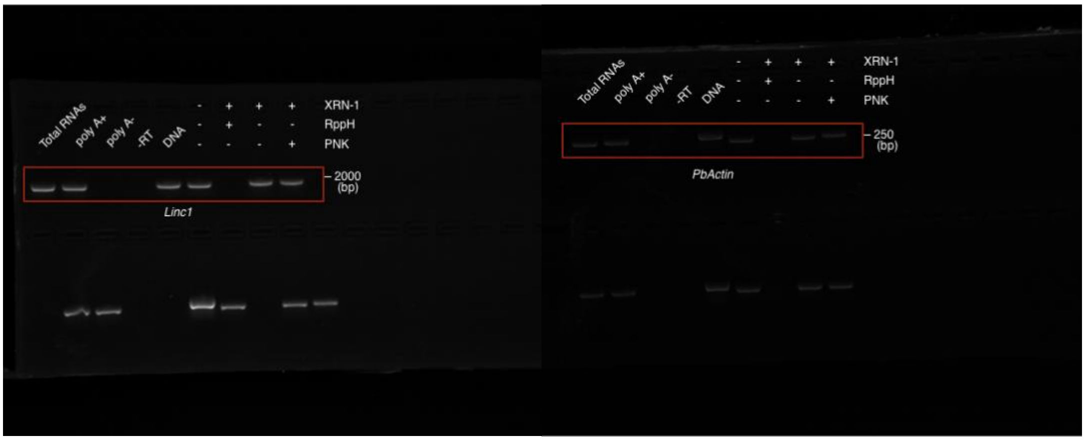

Figure 1e

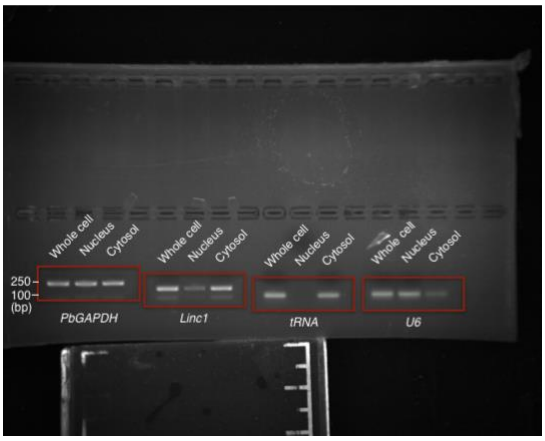

Figure 3d

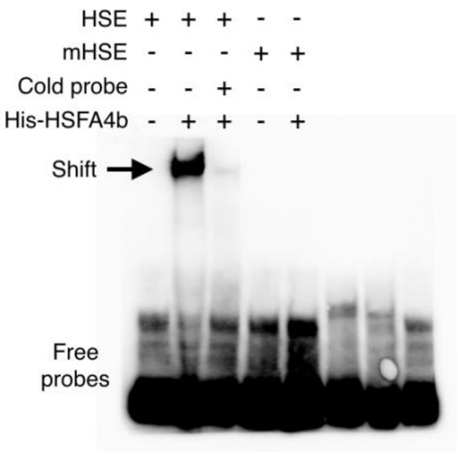

Figure 3e

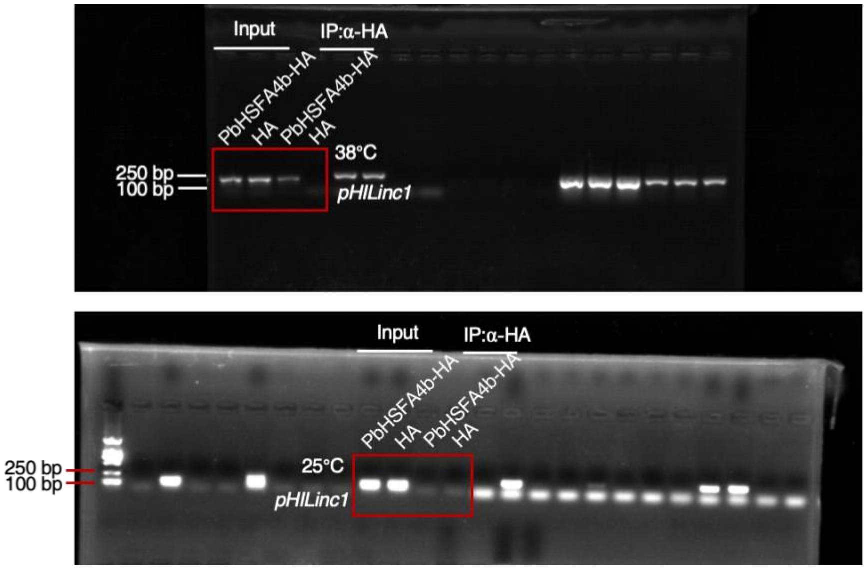

Figure 4d

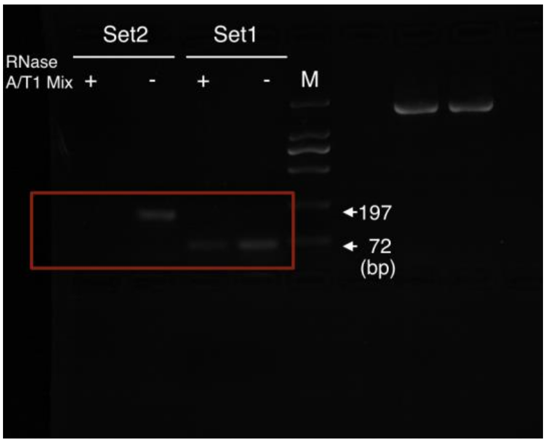

Figure 5c

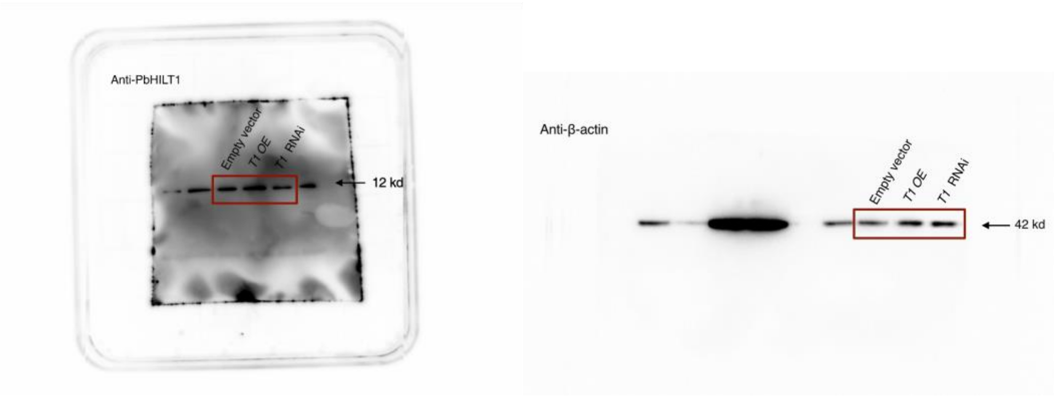

Figure 6b

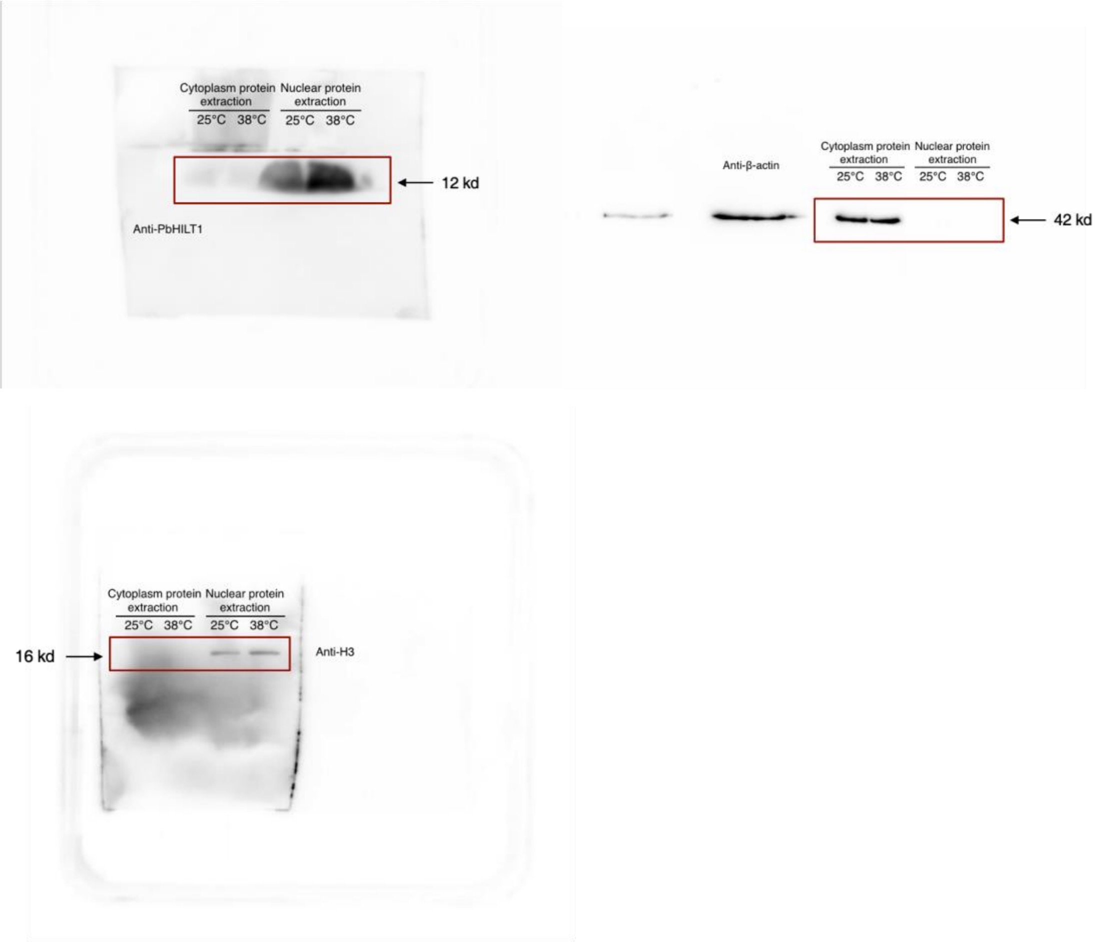

Figure 6d

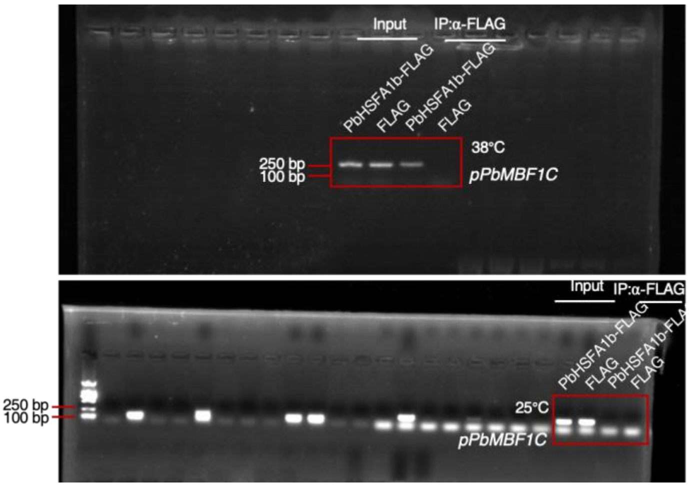

Figure 6f

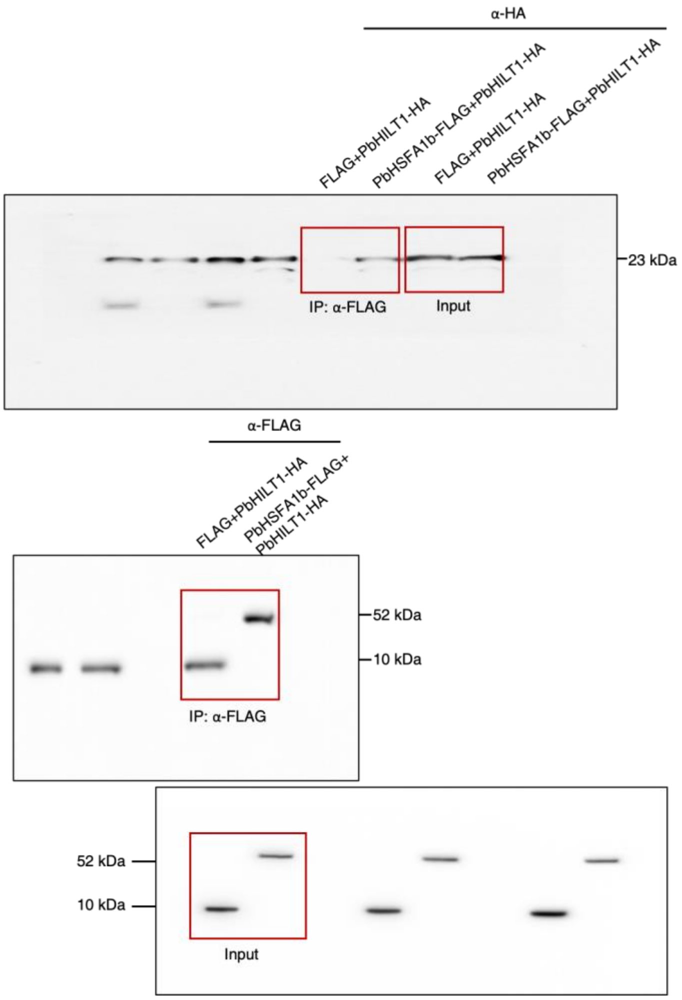

Supplementary Figure 4b

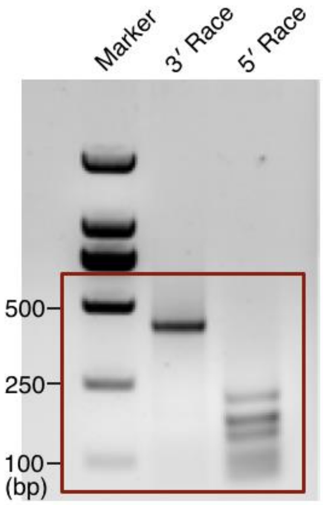

Supplementary Figure 10

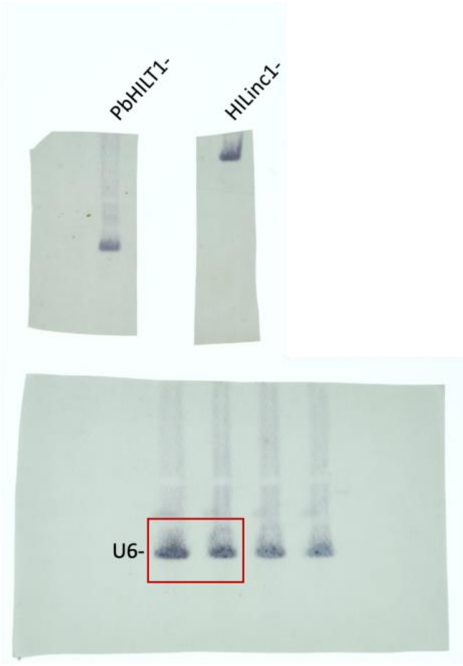

Supplementary Figure 18b

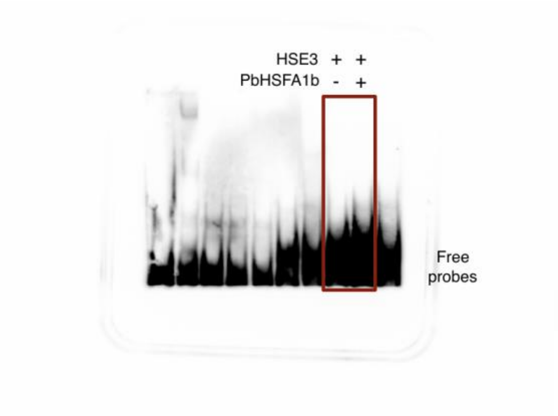

Supplementary Figure 18c

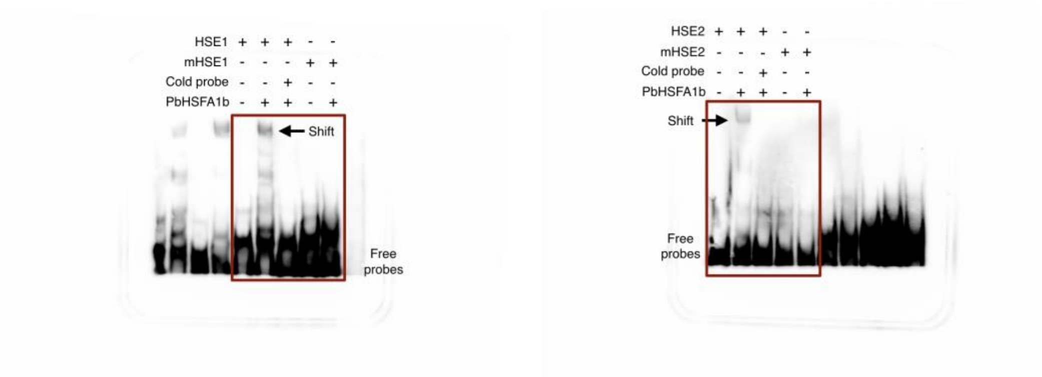

Supplementary Figure 20

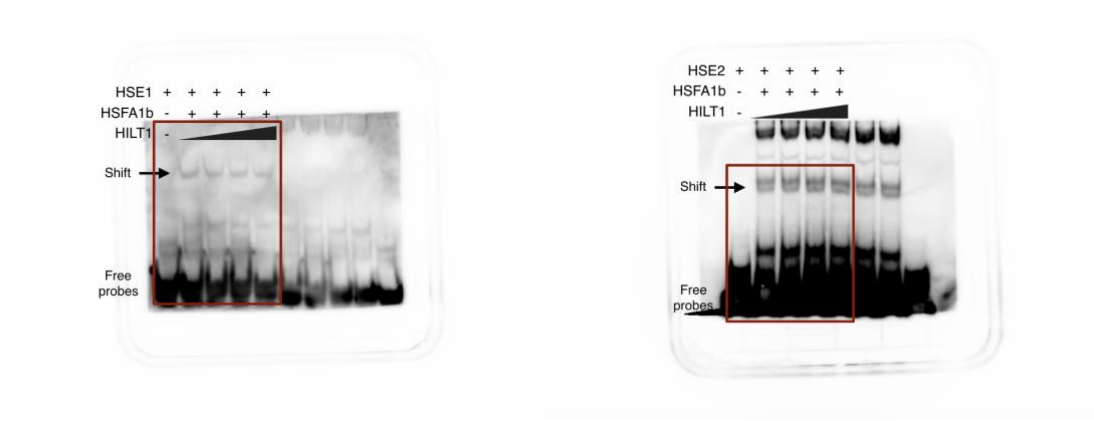

Supplement: Supplementary file 2 — Supplementary Information [file 42003_2022_4010_MOESM2_ESM.pdf]
