# Supplementary material for: A long noncoding RNA HILinc1 enhances pear thermotolerance by stabilizing PbHILT1 transcripts through complementary base pairing
Source: Commun Biol. 2022 Oct 26;5:1134. doi: 10.1038/s42003-022-04010-7 (PMC9606298; doi:10.1038/s42003-022-04010-7)
Supplement: Supplementary file 3 — Description of Additional Supplementary Files [file 42003_2022_4010_MOESM3_ESM.pdf]

Description of Additional Supplementary Files

File name: Supplementary Data 1

Description: Primers used in this study

File name: Supplementary Data 2

Description: The source data behind the graphs in the paper

File name: Supplementary Data 3

Description: Mass spectrometry-based proteomics data of semi-in vivo pulldown assay by PbHILT1-GST

File name: Supplementary Data 4

Description: LncRNAs differently expressed under 38 °C treatment

File name: Supplementary Data 5

Description: Mass spectrometry results of semi-in vivo pulldown assay by PbHILT1-GST
